# Analysis of selected fungi variation and its dependence on season and mountain range in southern Poland—key factors in drawing up trial guidelines for aeromycological monitoring

**DOI:** 10.1007/s10661-017-6243-5

**Published:** 2017-09-27

**Authors:** Wojciech Pusz, Ryszard Weber, Andrzej Dancewicz, Włodzimierz Kita

**Affiliations:** 1 Department of Plant Protection, Wroclaw University of Environmental and Life Sciences, Plac Grunwaldzki 24A, 50-363 Wroclaw, Poland; 2 Institute of Soil Science and Plant Cultivation—National Research Institute, ul. Orzechowa 62, 50-540 Wroclaw, Poland; 3 Institute of Meteorology and Water Managemen—National Research Institute, ul. Parkowa 30, 56-616 Wroclaw, Poland

**Keywords:** Aeromycological monitoring, Fungal diseases, Pathogenic fungi, Statistical methods

## Abstract

The aim of the study was to identify fungal spores, in particular plant pathogenic fungi, occurring in the air in selected mountain ranges. The results revealed not only the array of fungal species migrating with air currents from the Czech Republic and Slovakia but also how the season of the year affects the distribution of spores. Such studies may lay a foundation for future aeromycological monitoring, in accordance with the requirements for integrated plant protection. Aeromycological research was carried out between 2013 and 2016 at 3-month intervals in mountainous areas along the southern borders of Poland: the Bieszczady, the Pieniny, the Giant Mountains (*Karkonosze*) and the Babia Góra Massif. The research relied on impact method employing Air Ideal 3P sampler, which, by drawing in atmospheric air, also collects fungal spores. Regardless of altitudinal zonation, the changing weather conditions appeared to be the main reason for the variations in the number of the fungal spores under study in those years.

## Introduction

Fungi, present everywhere, including the air, are one of the elements of bioaerosol and are capable of surviving in this environment for an extended period of time (Dowd and Maier 1999). Airborne spores and mycelium are currently regarded as posing potential threat to humans suffering from allergies and may lead to a variety of health problems including asthmatic symptoms (Kurup et al. 2002; Asan et al. 2004). Understandably, one of the main objectives of aeromycological investigations is to confirm the presence of fungal spores which may adversely affect not only humans but also animals (Asan et al. 2004; Bugajny et al. 2005; Klarič and Pepeljnjak 2001; Topbas et al. 2006; Palmas and Cosentino 2009; Ianovici et al. 2011; Pusz et al. 2015). Fungi may additionally exert a detrimental influence on plants and spread infections (Meredith 1973; Dowd and Maier 1999; Jędryczka 2014). Furthermore, atmospheric air may carry parts of fungi which are capable of producing toxins, e.g. mycotoxins with possible negative impact on human and animal health (Raisi et al. 2012).

Consequently, some authors regard aeromycological studies as an element of an ‘early warning’ signalling plant disease (Dowd and Maier 1999; Pusz et al. 2013; Jędryczka 2014). Almaguer-Chávez and his team (2012) studied fungi concentrations colonising rice plantations and proved that aeromycological monitoring may be instrumental in signalling plant disease; this was corroborated by other researchers (Leyronas and Nicot 2012). Such studies may provide a database for mapping the distribution and/or occurrence of fungal spores which are both harmful to humans and pathogenic for plants (Toamssetti et al. 2011). This may be of profound importance in the case of the fungi which can be carried by wind over long distances (Palti and Cohen 1980; Leyronas and Nicot 2012) despite natural barriers such as high mountains (Nagarajan and Saharan 2007; Vaish et al. 2011; Pusz et al. 2013). Several authors thus reported, in mountainous areas, a number of fungal species pathogenic for plants. Klarič and Pepeljnjak (2001) identified airborne fungal spores of the genus *Cladosporium*, *Alternaria*, as well as *Fusarium*, *Sclerotinia* and *Botrytis* at 800–900 m above sea level, in the mountains near Zagreb, which was later confirmed by others (Pepeljnjak and Šegvič 2003; Magyar et al. 2012; Pusz et al. 2013). Aeromycological research in mountainous terrain constitutes unfortunately only an insignificant fraction of all aeromycological studies (Xia et al. 2012; Pusz et al. 2013).

The aim of this study was to determine the array of airborne fungal species, in particular those which are pathogenic for plants, occurring in selected mountain ranges. The results obtained during the study may be invaluable in establishing what fungi are carried with the air from the Czech Republic and Slovakia and how seasons of the year affect the distribution of fungal spores. Such analysis may in the future provide a springboard for aeromycological monitoring, which is in accordance with the statutory requirements imposed on integrated plant protection.

## Material and methods

**Fig. 1:**
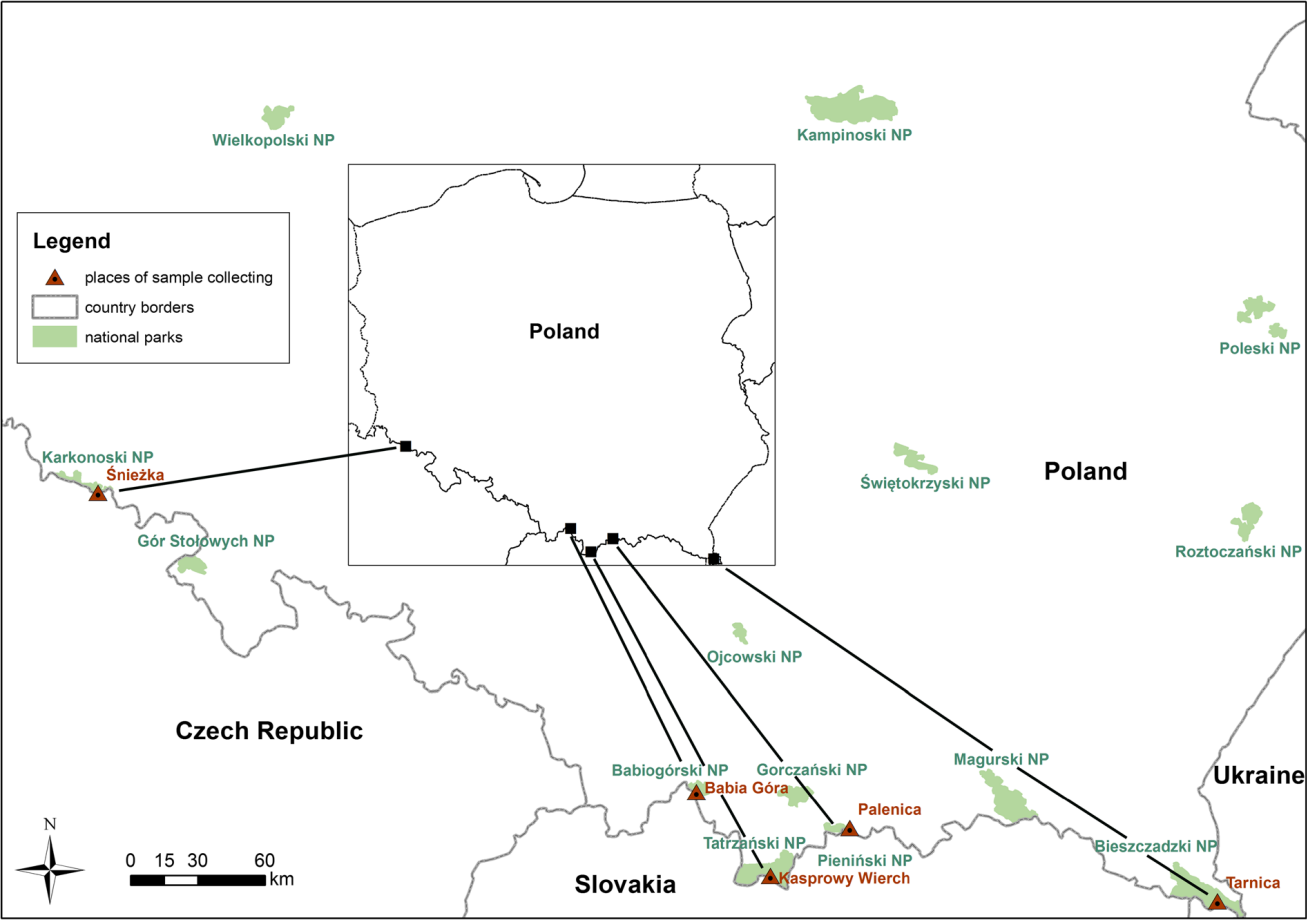
Sample collection site

The aeromycological studies were conducted at 3-month intervals in the years 2013–2016, close to Poland’s southern borders (Fig. 1). The precise locations were the following mountain ranges: the Bieszczady (Mount Tarnica 1346 m above sea level, 49° 04.483′ N, 22° 43.575′ E), the Pieniny (Mount Palenica 722 m, 49° 42.552′ N, 20° 48.066′ E), the Tatras (Mount Kasprowy Wierch 1997 m, 49° 13. 5461″ N, 19° 59′ 004′ E), the Babia Góra Massif (Mount Babia Góra 1725 m.n.p.m. (49° 34.392′ N, 19° 31.866′ E) and the Karkonosze (Mount Śnieżka—1603 m.n.p.m. (50° 44.172′ N, 15° 44.394′ E).

### Data collection

The research was performed employing impact method and using Air Ideal 3P. Drawing in the air, the device collects as well airborne fungal spores, which are then transferred onto petri dish and fed on growth medium potato dextrose agar (PDA, Biocorp.) with the addition of citric acid. Placed 1.5 m above ground, the device was set to collect 5, 100 and 150 l volume samples. All the measurements were repeated three times; each sample was deposited on nine petri dishes. These were stored at room temperature (20–22 °) over the period of 7 to 10 days. The next step to follow was to establish which species the grown fungi belonged to, and this was done on the strength of the morphological features defined by Pitt and Hocking (2009) and Watanabe (2011), and then to determine the number of colonies. The number of colonies grown on each dish was calculated for one cubic metre of air according to the formula

$$ X=\left(a\times 1000\right)/V $$

where *X* is the number of colony-forming units (CFUs) in 1 m^3^ of air, *a* is the total of fungal colonies which developed on petri dish from airborne spores, and *V* is the volume of drawn atmospheric air, expressed in litres.

### Statistical analyses

Out of 37 fungal species whose spores were identified in the mountainous belt of southern Poland, 12 most ubiquitous species were selected for the statistical analysis: *Alternaria alternata*, *Auerobasidium pullulans*, *Cladosporium cladosporioides*, *Cladosporium herbarum*, *Fusarium equiseti*, *Gliocladium catenulantum*, *Penicillium notatum*, *Penicillium purpurgenum*, *Penicillium waksmani*, *Penicillium vermiculatum*, *Sclerotinia sclerotiorium* and *Trichotecium roseum.* Log-linear analysis was performed to correlate the number of species under study, the year, the season and the mountains range. Some considerable disparities between the figures for the species under study and the expected figures, as revealed during the analysis, are indicative of a set of connections among the variables. The expected figures were converted from a logarithmic scale to a linear scale, yielding a model which may simply be based on a formula

$$ \mathrm{Ln}\left({E}_{ij}\right)=M+{\lambda_i}^X+{\lambda_j}^Y+{\lambda_{ij}}^{XY} $$

where *E*_*ij*_ is the expected frequency in cell, *M* is the general average based on equal cell count, *λ*_*i*_^*X*^ is the *it*h-row effect for variable *X*, *λ*_*j*_^*Y*^ is the *j*th-column effect for variable *Y*, and *λ*_*ij*_^*XY*^ is the interaction of *it*h-row effect and *j*th-column effect for variables *X* and *Y*.

The log-linear model allows a verification of the hypothesis predicting no interaction between two or more elements of the experiment. Discarding immaterial interactions, it also facilitates an examination of the impact of each factor on the changes occurring within the fungal population under study. The next step was to examine the patterns of the changing fungal species count in relation to the season of the year and mountain range; this was done by performing correspondence analysis. This analysis enables an accurate assessment of the way fungal species depend on other elements of the research and is instrumental in converting a 40D model, i.e. 2 years × 4 seasons × 5 mountains, to 2D graphs transferring comprehensively as much information on the changes as possible.

## Results

The weather conditions in the locations and the times of sampling are shown in Table 1. The temporary parameters of the ambient air may vary widely, which is perfectly explicable in terms of the extremely long distances between the locations. It is noteworthy that the collection of the data coincided almost invariably with the days when there was no precipitation, air humidity was often very low and so was wind speed, except the highest elevations: Mount Kasprowy Wierch and Mount Śnieżka.

**Table 1:** Weather conditions in the location where and at the time when the samples were collected

Number of sampling	Date	Hour	Fieldwork	Meteorological station	Temperature (°C)	Humidity (%)	Wind (m/s)	Precipitation (mm)
No. 1	22 July 2013	9.00	Tarnica	Wetlina	18	67–72	NW—1–2	
	22 July 2013	13.00	Palenica	Krościenko	23	40–45	NW—3–4	
	22 July 2013	15.00	Kasprowy Wierch	Kasprowy Wierch	13	68–71	N—3–4	
	23 July 2013	11.00	Babia Góra	Zawoja	20	58–63	NW—1–2	
	23 July 2013	17.00	Śnieżka	Śnieżka	13	55–60	N—7–9	
No. 2	06 November 2013	9.00	Tarnica	Wetlina	4–5	100	Wind—calm—1	
	06 November 2013	13.00	Palenica	Krościenko	6–7	75–80	Wind—calm—1	
	06 November 2013	15.00	Kasprowy Wierch	Kasprowy Wierch	− 6	100	NW—10–11	
	07 November 2013	11.00	Babia Góra	Zawoja	10	82–85	SW—1–2	Light rain
	07 November 2013	17.00	Śnieżka	Śnieżka	5	100	SW—17–20	Light rain
No. 3	12 January 2014	9.00	Tarnica	Wetlina	− 1	82–85	NW—2–3	
	12 January 2014	13.00	Palenica	Krościenko	1	84–87	NW—3–4	
	12 January 2014	15.00	Kasprowy Wierch	Kasprowy Wierch	− 10	90–93	NW—10–12	
	13 January 2014	11.00	Babia Góra	Zawoja	1–2	52–58	SE—1	
	13 January 2014	17.00	Śnieżka	Śnieżka	1–2	80–85	NW—9–19	
No. 4	05 April 2014	9.00	Tarnica	Wetlina	5	73–78	Wind—calm—1	
	05 April 2014	13.00	Palenica	Krościenko	11	55–60	NW—5–6	
	05 April 2014	15.00	Kasprowy Wierch	Kasprowy Wierch	2–3	98–100	SW—4–5	
	06 April 2014	11.00	Babia Góra	Zawoja	8	74–77	Wind—calm—1	
	06 April 2014	17.00	Śnieżka	Śnieżka	2	100	NW—11–12	
No. 5	30 June 2014	9.00	Tarnica	Wetlina	18	85–90	E—2–3	
	30 June 2014	13.00	Palenica	Krościenko	18	85–90	SE—1–2	
	30 June 2014	15.00	Kasprowy Wierch	Kasprowy Wierch	7–8	100	SE—6–8	Rain (storm)
	01 July 2014	11.00	Babia Góra	Zawoja	13	75–80	S—1	
	01 July 2014	17.00	Śnieżka	Śnieżka	6–7	90–95	W—3-6	
No. 6	07 November 2014	9.00	Tarnica	Wetlina	13	57–60	SE—4-5	
	07 November 2014	13.00	Palenica	Krościenko	16	64–67	SE—3–4	
	07 November 2014	15.00	Kasprowy Wierch	Kasprowy Wierch	5–6	95–100	SW—13–14	
	08 November 2014	11.00	Babia Góra	Zawoja	9	90–93	SW—1	Light rain
	08 November 2014	17.00	Śnieżka	Śnieżka	2–3	45–50	W—3–6	
No. 7	28 January 2015	9.00	Tarnica	Wetlina	− 4	90–95	Windless	
	28 January 2015	13.00	Palenica	Krościenko	2	63–64	SW—1	
	28 January 2015	15.00	Kasprowy Wierch	Kasprowy Wierch	− 11	91–92	SW—6–7	
	29 January 2015	11.00	Babia Góra	Zawoja	− 1	65–68	S—4–5	
	29 January 2015	17.00	Śnieżka	Śnieżka	− 9	92–95	SW—11–15	
No. 8	29 March 2015	9.00	Tarnica	Wetlina	1–2	87–90	NW—1–2	
	29 March 2015	13.00	Palenica	Krościenko	10	40–43	NW—2–3	
	29 March 2015	15.00	Kasprowy Wierch	Kasprowy Wierch	− 1	95–98	N—5–8	
	01 March 2015	11.00	Babia Góra	Zawoja	1	70–75	NW—2–3	Light rain
	01 March 2015	17.00	Śnieżka	Śnieżka	− 8	90–93	NW—28–38	

Two series of checks were conducted in 2013: in July, which is a summer month, and in November, which is an autumn month. In the summer, 14 species of fungi were observed in the Bieszczady Mountains, 7 in the Pieniny Mountains, 4 in the Tatras, 6 in the Beskidy Mountains and 8 in the Karkonosze Mountains. *Cladosporium cladosporoides*, which causes among others alternaria black spot, constituted the highest proportion of the fungi identified in all the locations, although other species pathogenic for plants were identified as well: *Botrytis cinerea*, *S. sclerotiorium*, *Rhizoctonia solani* and *A. alternata*.

In the autumn, 12 species were identified in the Bieszczady Mountains, 12 in the Pieniny Mountains, 7 in the Beskidy Mountains, 9 in the Tatras and 5 in the Karkonosze Mountains. The Bieszczady was mainly home to *Gliocladium catenulatum*; the same was true for the Pieniny, where additionally, *Cladosporium cladosporiodes* was observed, whereas in the sites, the most ubiquitous fungi were those of the genus *Cladosporium*.

In 2014, four series of checks were carried out; they spanned all four seasons of the year. January saw four species in the Bieszczady, where *C. cladosporioides* was the most popular; six species in the Pieniny, where *P. notatum* had the highest concentration among all the identified fungal spores; three in the Beskidy and one in the Karkonosze. No fungal colonies were observed in the Tatras at that time. In the spring, eight species were identified in the Bieszczady, where *A. alternata* and *C. cladosporioides* were most dominant, similarly as in the Pieniny, the Babia Góra Massif and the Karkonosze; eight species were observed in the Pieniny; two in the Tatras, where the most widespread species was *G. catenulatum*; five in the Beskidy and four in the Karkonosze Mountains.

The summer months saw nine species of airborne fungi in the Bieszczady, six in the Pieniny, six in the Tatras, four in the Beskidy and six in the Karkonosze. In the Bieszczady, *Acremonium strictum* and *B. cinerea* made up the highest percentage of the airborne fungi. The Pieniny results showed a similar trend as that in the spring, with *A. alternata* and *C. cladosporioides* dominating the samples; these had the same levels of concentration in the Tatras. The Babia Góra Massif was home predominantly to *B. cinerea* airborne spores, while *P. notatum* and *C. cladosporioides* were the most numerous airborne spores in the Karkonosze. The last sample collection was performed in the autumn: 11 airborne species were identified in the Bieszczady, 10 in the Pieniny, 10 in the Tatras, 9 in the Beskidy and 10 in the Karkonosze. The highest share was claimed by *C. cladosporioides* in the Bieszczady and the Pieniny, *G. catenulatum* in the Tatras, this and *A. strictum* in the Babia Góra Massif and *C. cladosporioides* in the Karkonosze.

The last year of the research witnessed only two sample collections, in the winter and in the spring. The winter data revealed six species in the Bieszczady, four in the Tatras, two in the Babia Góra Massif, two in the Pieniny and two in the Karkonosze. The spring data, on the other hand, revealed 6 species of airborne fungi in the Bieszczady, 5 in the Tatras, 8 in the Pieniny, 10 in the Babia Góra Massif and 4 in the Karkonosze. In the winter, the most numerous airborne fungal spores were those of the genus *Cladosporium*, with the exception of the Bieszczady, where *A. strictum* was the most dominant. In the spring, in the Tatras and the Babia Góra Massif, *C. cladosporioides* took over from other fungi, as did *G. catenulatum* in other locations.

The analysis of the optimum statistical model assessing the influence of years, seasons and mountain range on the concentrations of fungi was performed by applying chi-squared test to the main results as well as to the many interactions among the various elements of the experiment. The computed statistics for the second-order and third-order model showed considerable quantities; hence, the hypothesis that there is no correlation between the number of fungal spores under study and when (year and season) as well as where (mountain range) the samples were collected had to be rejected at level *p* < 0.001. The data revealed considerable fluctuations in the number of fungus species occurring in the mountainous sites under study in different years and seasons, which proves that atmospheric conditions exert a massive impact on the concentrations of the analysed isolates (Table 1). Strong interactions between fungus species and location or time are confirmed by the variations in the number of species identified in spring, summer, autumn and winter across the five mountain ranges.

Tables 2, 3 and 4 show clearly that the seasons at the turn of 2014 were special in terms of a markedly elevated count of fungal spores under study, compared with the summer and autumn of 2014 and with the winter and spring of 2015. The years 2013 and 2014 saw *C. cladosporioides* as the dominant species, regardless of location; *A. alternata* and *G. catenulantum* followed suit. The Babia Góra Massif was a site boasting a remarkably higher number of spores under study, whereas Mount Śnieżka offered the least favourable conditions for the reproduction of these fungi. Looking at Tables 5 and 6, one can easily notice a rich diversity of the ecosystem in the years when samples were taken: the summer of 2013 was more conducive to a rapid multiplication of spores than the spring, autumn or winter of 2014 (Table 5). Conversely, the weather conditions in the autumn of 2014 were much more favourable for the population in question than those in the summer of that same year, or in the winter and spring of 2015 (Table 6).

**Table 2:** Tests of the main effects, marginal and partial associations and the interactions between experiment factors

Effect	Degrees of freedom	Chi^2^ partial association	Significant level (*p*)	Chi^2^ marginal association	Significant level (*p*)
Mountains (1)	4	5388.59	< 0.001	5388.59	< 0.001
Years (2)	1	109.02	< 0.001	109.02	< 0.001
Seasons (3)	3	11,075.71	< 0.001	1175.71	< 0.001
Fungus species (4)	11	30,558.43	< 0.001	30,558.43	< 0.001
1 × 2	4	1261.0512	< 0.001	1261.05	< 0.001
1 × 3	12	2434.02	< 0.001	2250.50	< 0.001
1 × 4	44	7875.94	< 0.001	8171.86	< 0.001
2 × 3	3	2081.48	< 0.001	3065.23	< 0.001
2 × 4	11	1449.21	< 0.001	2912.79	< 0.001
3 × 4	33	7276.98	< 0.001	8827.68	< 0.001
1 × 2 × 3	12	1279.56	< 0.001	3232.11	< 0.001
1 × 2 × 4	44	2594.12	< 0.001	5371.04	< 0.001
1 × 3 × 4	132	4569.02	< 0.001	5095.57	< 0.001
2 × 3 × 4	33	1694.28	< 0.001	18,995.92	< 0.001

**Table 3:** Fungus isolates’ count in relation to the type of mountain rage in the years 2013–2014

Fungus species	Sample collection site					Aggregates
	Śnieżka	Palenica	Babia Góra	Kasprowy	Tarnica	
*Alternaria alternata*	3	49	294	813	146	1305
*Auerobasidium pullulans*	16	13	3	14	4	50
*Cladosporium cladosporioides*	120	1752	3974	99	453	6398
*Cladosporium herbarum*	101	3	4	276	128	512
*Fusarium equiseti*	3	3	8	4	15	33
*Gliocladium catenulantum*	23	422	4	48	242	739
*Penicillium notatum*	45	122	62	12	30	271
*Penicillium purpurgenum*	3	2	3	3	41	52
*Penicillium waksmani*	9	74	4	14	68	169
*Penicillium vermiculatum*	16	16	253	4	43	332
*Sclerotinia sclerotiorium*	8	82	16	4	28	138
*Trichotecium roseum*	3	27	3	3	4	40
aggregates	350	2565	4628	1294	1202	10,039

**Table 4:** Fungus isolates’ count in relation to the type of mountain rage in the years 2014–2015

Fungus species	Sample collection site					Aggregates
	Śnieżka	Palenica	Babia Góra	Kasprowy	Tarnica	
*Alternaria alternata*	48	200	55	882	75	1260
*Auerobasidium pullulans*	2	41	9	3	116	171
*Cladosporium cladosporioides*	413	790	426	485	759	2873
*Cladosporium herbarum*	4	114	29	10	221	378
*Fusarium equiseti*	16	48	128	75	338	605
*Gliocladium catenulantum*	15	74	1946	82	142	2259
*Penicillium notatum*	160	73	16	23	49	321
*Penicillium purpurgenum*	23	35	28	16	3	105
*Penicillium waksmani*	49	75	23	9	76	232
*Penicillium vermiculatum*	56	29	9	10	4	108
*Sclerotinia sclerotiorium*	30	42	17	83	75	247
*Trichotecium roseum*	4	61	17	21	43	146
Aggregates	820	1582	2703	1699	1801	8605

**Table 5:** Fungus isolates’ count in relation to the season in the years 2013–2014

Fungus species	Seasons of the year				Aggregates
	Summer	Autumn	Winter	Spring	
*Alternaria alternata*	1173	34	2	96	1305
*Auerobasidium pullulans*	28	14	3	5	50
*Cladosporium cladosporioides*	5183	567	89	559	6398
*Cladosporium herbarum*	221	283	5	3	512
*Fusarium equiseti*	5	3	3	22	33
*Gliocladium catenulantum*	4	682	4	49	739
*Penicillium notatum*	37	17	110	107	271
*Penicillium purpurgenum*	24	22	4	2	52
*Penicillium waksmani*	31	26	16	96	169
*Penicillium vermiculatum*	82	230	4	16	332
*Sclerotinia sclerotiorium*	30	42	4	62	138
*Trichotecium roseum*	30	4	4	2	40
aggregates	6848	1924	248	1019	10,039

**Table 6:** Fungus isolates’ count in relation to the season in the years 2014–2015

Fungus species	Seasons of the year				Aggregates
	Summer	Autumn	Winter	Spring	
*Alternaria alternata*	973	247	35	5	1260
*Auerobasidium pullulans*	5	14	24	28	71
*Cladosporium cladosporioides*	962	1226	399	286	2873
*Cladosporium herbarum*	198	43	89	48	378
*Fusarium equiseti*	177	331	1	96	605
*Gliocladium catenulantum*	3	2008	16	232	2259
*Penicillium notatum*	116	166	5	34	321
*Penicillium purpurgenum*	5	81	18	1	105
*Penicillium waksmani*	5	162	36	29	232
*Penicillium vermiculatum*	17	51	24	16	108
*Sclerotinia sclerotiorium*	64	174	4	5	247
*Trichotecium roseum*	120	4	4	18	146
aggregates	2645	4507	655	798	8605

**Fig. 2:**
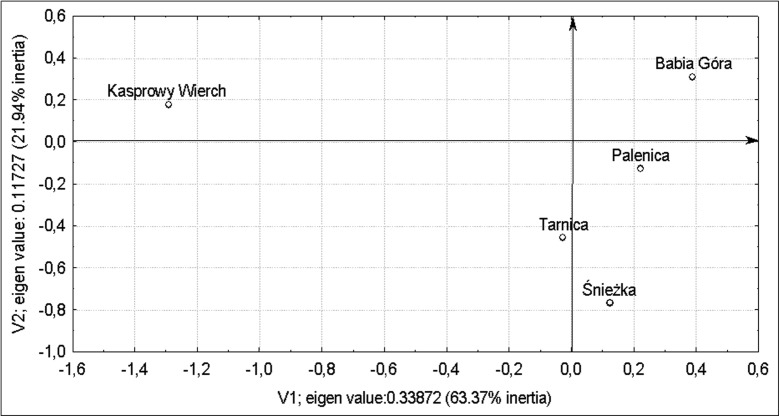
Correspondence analysis of the fluctuations in the population of fungi in each mountain range

In the next part of the paper, we tried to discern regular patterns in the way the factors under study, i.e. year, season and mountain’s environment, affect the levels of concentration of the fungi. This was done by correspondence analysis used routinely by Hill (1974), which is frequently referred to as reciprocal averaging or optimal scaling. The 2D graphs presented in this work successfully transferred, from the original multi-dimensional system, 85.31% of the total of the environmental variables in the mountain peaks under study. The arrangement of mountain peaks representing individual environments is indicative, on closer inspection, of a pattern where ‘Kasprowy Wierch’ (the Tatras) is located on the left-hand side of the coordinates (Fig. 2) and constitutes a separate one-element group which differs fundamentally, with regard to the fluctuations in the numbers of the 12 fungal spores under study, from the other group of the mountains, namely ‘Palenica’ (the Pieniny), ‘Tarnica’ (the Bieszczady) and ‘Śnieżka’ (the Karkonosze). The environmental conditions in Kasprowy Wierch were conducive to a more rapid multiplication of *A. alternata*, compared with the other mountain peaks. A great distance between the points representing ‘Babia Góra’ and Śnieżka confirms that the fluctuations in the concentrations of the 12 fungus species under study embrace a rich diversity of patterns. The atmospheric conditions around Babia Góra were instrumental in the substantial increase in the number of the spores, which was not true for Śnieżka.

**Fig. 3:**
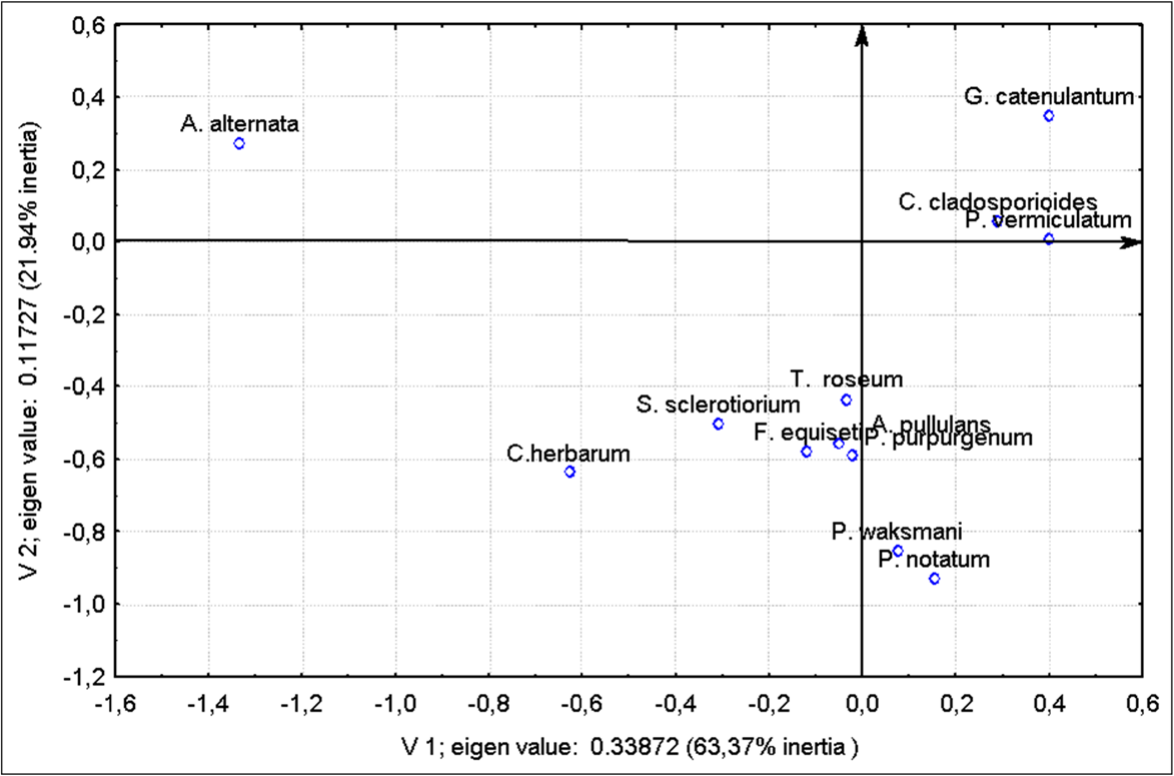
Correspondence analysis of the variations in each fungus species in relation to the type of mountain range and season of the year

The analysis of the variations within the fungus population, attributable to the year, season or the mountain range, reveals three groups of species which differ enormously from one another (Fig. 3). The first, one-element, group is situated at a considerable distance from other groups and is formed by *A. alternata*. Compared with other fungi, this one exhibits quite a different pattern of fluctuations in the spore count for each season, year or mountain range. Another group includes *G. catenulantum*, *C. cladosporioides* and *P. vermiculatum*, in which the fluctuations in the count of the spores follow a more or less uniform pattern. The last group comprises *T. roseum*, *S. sclerotiorium*, *C. herbarium*, *F. equiseti*, *P. purpurgenum*, *P. waksmani*, *A. pullulans* and *P. notatum* and is more diffuse.

## Discussion

In this day and age, when the changes affect climate and habitat, and when habitat and the environment are irreversibly fragmented, it is imperative that we undertake environmental research studies to forecast, to a lesser or greater extent, the changes occurring in nature (Smith 1994; de Groot et al. 1995). One such group of organisms, which is becoming more and more crucial in the environmental monitoring and human biomonitoring, is fungi (Porca et al. 2011; Jędryczko 2014; Kokurewicz et al. 2016; Pusz 2016).

Fungi play a prominent role in economy and environmental health. Plant pathogens are known not only to contribute to a poor crop but also to produce mycotoxins harmful to humans and animals alike (Jędryczko 2014; Gołębiowska et al. 2016). In the spirit of integrated plant protection (Norris et al. 2003), aeromycological monitoring can be justifiably required not only in agriculture (Jędryczko 2014) or forestry (Clarke 1995) but also in protected areas (Pusz et al. 2013), where plant diseases may pose threat to rare and endangered species (Pusz 2016). It is therefore vital that the rules for permanent aeromycological monitoring, especially in extreme environments such as mountainous areas, are drawn up taking into account the rapidly changing atmospheric conditions (Bonello and Blodgett 2003; Pusz et al. 2013).

In this research, the highest numbers of fungus species were observed in summer and autumn, which is corroborated by other authors (Asan et al. 2004; Almaguer-Chávez et al. 2012). The most dominant fungus in all the locations was *C. cladosporioides*. The fungus is popularly classified as a truly cosmopolitan organism, fairly flexible in terms of temperature but exhibiting a preference for higher air humidity accompanied by lower temperatures. It usually develops as a saprotroph deriving nourishment from dead plants. It may also turn pathogen par excellence for several living plants (Ogórek et al. 2012). The fungi belonging to the genus *Cladosporium* frequently colonise the seeds of many other plants, a fact confirmed by several authors (Fakhrunnisa and Ghaffar 2006; Pusz 2009).

The typical plant pathogens were strongly represented by the fungi of the genera *Fusarium* and *S. sclerotiorium*. The former pose an acute problem not only for agriculture, as they infect cereals and other cultivated plants (Gołębiowska et al. 2016), but also for plants growing wild (Pusz 2016). They may additionally colonise dead plants and thus constitute threat to farm animals (Pusz 2016). Another threat is posed by mycotoxins present in plant products as well as in the air (Cabral 2012). By contrast, *S. sclerotorium*, which causes white mould, is a polyphage which severely affects a great number of plants (Bolton et al. 2006). The presence of these species in the bioaerosol was confirmed also in the work of Pusz et al. (2013). By tracking the fluctuations in the levels of CFU, it may become feasible to predict their impact on both agricultural crops and woodland or ornamental tree nurseries. Atmospheric conditions such as wind and also temperature or precipitation should be additionally taken into account; rain appears to be, together with wind, one of the key factors affecting the concentration levels of airborne fungal spores (Jones and Harrison 2004). It is a salient fact that precipitation is likely to lead to a raised spore count, including plant pathogens (Huffman et al. 2013).

Another issue to address when establishing guidelines for aeromycological monitoring is the method of sample collection. A very common one relies on the Hirst apparatus, which draws the air in for a number of days, e.g. seven, at a fixed volume, e.g. 10 l, at an interval of several dozen minutes. This is followed by the removal of the tape with the spores, to count them and identify the species (Jędryczka et al. 2008; Jędryczka 2014). Another method, called impact method, uses likewise a variety of mechanical devices to draw in the air: in this case, the spores are deposited directly on petri dish containing solidified growth medium. The changing size of the fungi colony becomes visible after a couple of days (Pusz et al. 2013; Jędryczka 2014). Each method has its benefits and drawbacks. The biggest advantage of Hirst apparatus is that the observations of airborne spores can be extended over a prolonged period of time, but at the same time, the system suffers from the disadvantage of pollen, small insects and other impurities finding their way onto the tape. Conversely, the impact method offers a picture of what arrives on the dish only at the moment of taking the sample; however, the selective growth medium nourishes solely the organisms, e.g. fungi, whose concentration levels we are interested in. Furthermore, we can determine the viability of the spores and mycelium: observations which Hirst apparatus cannot facilitate in the same way (McCartney et al. 1997; Calderon et al. 2002).

Summing up, there is evidence to suggest that a systematic aeromycological monitoring, as used in agriculture and forestry or to forecast the presence of allergens, should include atmospheric conditions in the area under study, in particular wind direction and the average wind speed. Precipitation and air humidity, too, should be taken into account when designing the algorithms. Another significant factor to consider is a careful choice of the method: with the pros and cons of each, it is, at this stage, a challenging task. The use of Hirst apparatus requires unarguably regular sample collections by applying impact method to determine precisely the viability of the spores and assess their potential threat to plants. Another demanding task is to devise effective statistical methods that would allow the available data to reflect faithfully the state of the bioaerosol and would develop adequate prognostic tools. This, however, calls for further in-depth research.

## Conclusions

Regardless of location, the ever-changing weather patterns in each year were the primary cause of the fluctuations in the numbers of fungal spores under study. The atmospheric conditions in the years 2014–2015 were more conducive to the reproduction and growth of *G. catenulantum*. The reverse was true for the years 2013–2014, when a significantly greater number of *C. cladosporioides* spores were identified, compared with the summer and autumn of 2014 and the winter and spring of 2015. The differences in the biodiversity and climate of the mountains in southern Poland were also the reason why the concentrations of fungal spores under study varied enormously. The conditions in the Karkonosze Mountains were less favourable for the fungi than the conditions in the Babia Góra Massif. Correspondence analysis revealed that the atmospheric conditions in the locations covered by the research affected *A. alternata* to a varying degree, in contrast to other species. This seems to confirm that factors such as habitat or topo-climatic conditions (especially like precipitation, temperature and wind speed) have a profound effect on the results obtained in the study and that they should be rigorously followed when laying down guidelines for aeromycological monitoring.
